# Predictors of Attrition and Immunological Failure in HIV-1 Patients on Highly Active Antiretroviral Therapy from Different Healthcare Settings in Mozambique

**DOI:** 10.1371/journal.pone.0082718

**Published:** 2013-12-20

**Authors:** Claudia Palladino, Verónica Briz, José María Bellón, Inês Bártolo, Patrícia Carvalho, Ricardo Camacho, M. Ángeles Muñoz-Fernández, Rui Bastos, Rolanda Manuel, José Casanovas, Nuno Taveira

**Affiliations:** 1 Centro de Patogénese Molecular, Unidade dos Retrovírus e Infecções Associadas, Faculdade de Farmácia, Universidade de Lisboa, Lisbon, Portugal; 2 Laboratorio de Inmuno-Biología Molecular, Hospital General Universitario “Gregorio Marañón”, Networking Research Center on Bioengineering, Biomaterials and Nanomedicine (CIBER-BBN), Madrid, Spain; 3 Unidad de Investigación, Fundación para la Investigación Biomédica, Hospital General Universitario “Gregorio Marañón”, Madrid, Spain; 4 Laboratório de Biologia Molecular, Centro Hospitalar de Lisboa Ocidental, Lisbon, Portugal; 5 Serviço de Dermatologia e Venereologia, Hospital de Dia, Hospital Central de Maputo, Maputo, Mozambique; 6 Unidade de Imunodiagnóstico Viral, Departamento Académico de Microbiologia, Faculdade de Medicina, Universidade Eduardo Mondlane, Maputo, Mozambique; 7 Centro de Investigação Interdisciplinar Egas Moniz (CiiEM), Instituto Superior de Ciências da Saúde Egas Moniz, Caparica, Portugal; Rush University, United States of America

## Abstract

In Mozambique, the evaluation of retention in HIV care and ART programmes is limited. To assess rate and predictors of attrition (no retention in care) and HAART effectiveness in HIV-1 infected patients who pay for medication and laboratory testing in Mozambique, we conducted a multicenter survey of HIV-1-infected patients who started HAART during 2002–2006. Cox proportional hazard models were used to assess risk of attrition and of therapy failure. Overall, 142 patients from 16 healthcare centers located in the capital city Maputo were followed-up for 22.2 months (12.1–46.7). The retention rate was 75%, 48% and 37% after one, two and three years, respectively. Risk of attrition was lower in patients with higher baseline CD4 count (P = 0.022) and attending healthcare center 1 (HCC1) (P = 0.013). The proportion of individuals with CD4 count ≤200 cells/µL was 55% (78/142) at baseline and decreased to 6% (3/52) at 36 months. Among the patients with available VL, 86% (64/74) achieved undetectable VL levels. The rate of immunologic failure was 17.2% (95% CI: 12.6–22.9) per 100 person-years. Risk of failure was associated to higher baseline CD4 count (P = 0.002), likely reflecting low adherence levels, and decreased with baseline VL ≥10,000 copies/mL (P = 0.033). These results suggest that HAART can be effective in HIV-1 infected patients from Mozambique that pay for their medication and laboratory testing. Further studies are required to identify the causes for low retention rates in patients with low CD4 counts and to better understand the association between healthcare setting and attrition rate.

## Introduction

Mozambique suffers from one of the highest HIV/AIDS burdens in the world, with HIV-1 prevalence of 21% in the southern regions where the capital city Maputo is located [1]. Since mid-1990s antiretroviral therapy (ART) has been available in Maputo in healthcare settings for patients with medium-high socioeconomic status, before national ART program was implemented. These patients can afford to pay for their medication, which are provided by health practitioners, and so they have access to antiretrovirals different from those being currently given to the general population. In Kenya and Uganda, treatment interruption and drug resistance levels are higher among HIV patients that pay for their own medication and laboratory testing compared to patients on free-ART programs suggesting that new strategies may be needed to improve treatment outcomes in this population [2], [3]. At this time, no studies have evaluated the response to ART among this particular population of patients who attend the pay-for-care setting in Mozambique. Moreover, the issue of retention in HIV care programmes has never been addressed in this special group. The current study was conducted to evaluate the determinants of attrition (no retention in care) after the initiation of ART in HIV-1-infected patients undergoing self-paid medication and laboratory testing in Maputo, Mozambique, and to assess the effectiveness of ART and the predictors of immunologic failure in this population.

## Patients and Methods

### Ethics Statement

The study was conducted according to the Declaration of Helsinki with the approval of the ethics committee of the Faculty of Medicine (Eduardo Mondlane University, Maputo). Written informed consent was obtained from all participants.

### The Maputo HIV-1 Cohort

The Maputo HIV-1 Cohort was established to better describe the epidemiology of HIV-1 infection and care patterns and treatment among patients with medium-high socioeconomic status who self-paid laboratory analyses and ART. The Cohort prospectively enrolled antiretroviral-naïve HIV-1-infected patients recruited in 16 healthcare centers throughout the Maputo province. All patients aged ≥18 years at ART initiation, with documented date of start of highly active antiretroviral therapy (HAART) comprised of at least 3 antiretrovirals, with baseline CD4 T-cell count (for the six months before HAART initiation) and with follow-up of ≥3 months were eligible for this retrospective survey. The HIV-1 infection was determined on serum or plasma samples by Uni-Gold Recombigen (Trinity Biotech, Bray, Ireland) and Determine HIV-1/2 (Abbott, Bedford, UK) assays. Immune cell counts were determined by flow cytometry (FACSCalibur, Becton Dickinson, NJ) and plasma HIV-1 RNA (viral load, VL) by COBAS Amplicor test v1.5 (Roche, Mannheim, Germany), which has a detection limit of 400 copies/mL. Resistance genotyping was performed by ViroSeq HIV-1 Genotyping System (Abbott, IL).

### Statistical Analysis

Baseline characteristics were reported with medians and interquartile range (IQR) for continuous variables and percentages for categorical variables. Kolmogorov-Smirnoff test was used to evaluate the distribution of variables. Fisher exact test or χ^2^ were used to compare categorical variables. The median of HIV-1 markers and biochemical parameters was calculated for each patient per semester. A window period of 6 months was allowed for measurements at each time-point. A median for the study population was then calculated to evaluate the evolution of each marker (Wilcoxon tests). Rates of change (slopes) in total lymphocyte count (TLC), CD4 and CD8 counts were calculated from 3 consecutive measurements (Deming regression). Differences in slopes among groups according to different baseline CD4 count were analyzed (Kruskal-Wallis and Mann-Whitney U tests) and correlation between slopes was assessed (Spearman Rank test). The risk of attrition (no retention in care) after HAART initiation and the risk of immunologic failure over time were estimated by survival analyses using Kaplan-Meier curves and Cox proportional hazards models. Time was calculated from the date of HAART initiation to the outcome event. Data were censored on the last visit date for individuals not retained in care. Information on vital status (alive or death) for those not retained in care was not available, thus the definition of outcome measure of attrition from care combined both patients who died and those lost to follow-up, who had failed to return for treatment for 3 months after their scheduled visit for reasons unrelated to death. Immunologic failure was defined as: fall of CD4 count to baseline (or below) or 50% fall from on-treatment peak value or persistent CD4 levels below 100 cells/µL [4]. Time to virologic suppression was estimated by survival analyses for the following endpoints: VL ≤400 copies/mL; a reduction of VL >1 log_10_; a reduction of VL >2 log_10._ SPSS (v.17; SPSS Inc., Chicago, IL) and GraphPad Prism (v.4; San Diego, CA) were used. Statistical significance was defined as P<0.05 (2-tailed).

## Results

### Baseline Characteristics

Overall, 142 patients from 16 healthcare centers (HCC) who started HAART between 2002 and 2006 were eligible. Most patients (62, 43.7%) were from a centrally located clinic (HCC1); the remaining patients were from 15 small private clinics located around Maputo. For convenience of analysis these clinics were grouped into one and named HCC2. The median follow-up was 22.2 months (12.1–46.7). The median baseline CD4 count was 185 cells/µL (126–290). Baseline VL was available in 54% (76/142) of patients, who had a median of 5.3 (4.8–5.9) log_10_ copies/mL, and was inversely correlated to CD4 counts (r = −0.313; P = 0.006). Median CD4 count and age were similar between patients with available and unavailable VL. Median CD4 count, VL and age were similar between patients attending HCC1 and HCC2. Other baseline characteristics and ART use are listed in Table S1.

### The Risk of Attrition is Associated with Low Baseline CD4 Count and the Healthcare Center

The retention rate was 75%, 48% and 37% after one, two and three years, respectively (Figure S1). Risk of attrition from HIV care after initiation of HAART decreased at higher baseline CD4 count (AHR: 0.832; 95% CI: 0.70–0.97; P = 0.022) and was related to the HCC of origin such that patients attending HCC2 had a significant higher risk of attrition than those attending HCC1 (AHR: 1.75; 95% CI: 1.13–2.71; P = 0.013) (Table 1).

**Table 1:** Analyses of risk of attrition from HIV care in the study population.

	Attrition			
Analysis, factor	N.	N. of cases (%)	Hazard ratio (95% CI)	P
**Univariate**				
Age at first regimen start^a^	142	90 (63.4)	0.99 (0.90–1.10)	.87
Sex				
Men	82	50 (61.0)	1	
Women	60	40 (66.7)	1.34 (0.88–2.03)	.17
Ethnicity				
Other	27	15 (55.6)	1	
Black	108	69 (63.9)	1.13 (0.65–1.99)	.66
Baseline CD4 count^b^	142	90 (63.4)	0.82 (0.69–0.96)	**.** ***016***
Baseline log_10_ HIV-1 RNA				
<10,000	8	4 (50.0)	1	
≥10,000	68	45 (66.2)	1.30 (0.47–3.62)	.61
Unknown	66	41 (62.1)	1.05 (0.37–2.93)	.93
First-line regimen^c^				
PI-based	16	8 (50.0)	1	
NNRTI-based	125	81 (64.8)	1.57 (0.76–3.26)	.23
Healthcare center				
HCC1	62	34 (54.8)	1	
HCC2	80	56 (70.0)	1.80 (1.16–2.79)	**.** ***009***
**Multivariable**				
Baseline CD4 count^b^	142	90 (63.4)	0.83 (0.70–0.97)	**.** ***022***
Healthcare center				
HCC1	62	34 (54.8)	1	
HCC2	80	56 (70.0)	1.75 (1.13–2.71)	**.** ***013***

Legend: Adjusted hazard ratios (AHR) were derived from a standard Cox proportional hazard model. CI, confidence intervals; NNRTI, nonnucleoside reverse-transcriptase inhibitor; PI, protease inhibitor; HCC, healthcare center.

^a^ Per 5-y increase,

µL,^b^ per 100 cells/

+1NNRTI +1PI as initial HAART regimen.^c^ excludes one patient who was treated with 1NRTI

### The Risk of Immunologic Failure is Associated with Baseline CD4 Count and Viral Load

**Figure 1:**
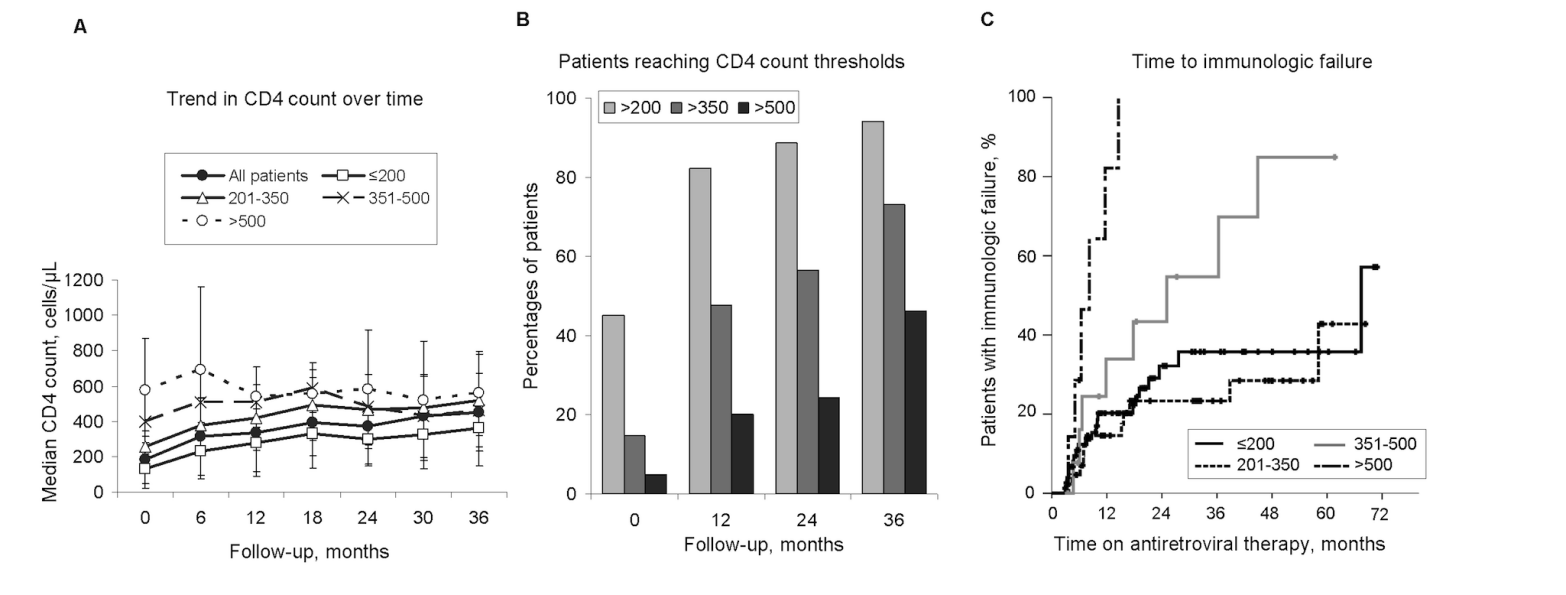
Immunological response to HAART during follow-up. Legend: A) Median increase in CD4+ T-cell count during the first 3 years of follow-up according to baseline CD4 T-cell count in the study population; dots represent median values over the study period by CD4 T-cell count baseline strata; bars represent interquartile range; B) Percentage of patients reaching clinically relevant thresholds of CD4 T-cell count (>200 cells/µL; >350 cells/µL; >500 cells/µL) during follow-up; C) Kaplan-Meier plots of the probability of immunologic failure according to baseline CD4 T-cell count (cells/µL) during follow-up.

Median CD4 count increased with time on HAART with a rapid average increase [113 cells/µL (38–187)] during the first semester (P<0.001) (Table S2, Figure 1A). Consistently, whereas the proportion of individuals with CD4 count ≤200 cells/µL was 55% (78/142) at baseline, this proportion decreased to 6% (3/52) at 36 months, while those with CD4 count >500 cells/µL increased from 5% (7/142) to 46% (24/52) (Figure 1B). Patients who had no available CD4 count at 36 months presented higher immunosuppression at baseline than patients with available data (169 cells/µL [111–248] vs 248 [153–329]; P = 0.005). CD8 count showed small changes over time, while CD4/CD8 ratio increased to 0.5 (0.4–0.7) at 36 months (P<0.001) (Figure S2A–B). The rate of immunologic failure was 17.2% (95% CI: 12.6–22.9) per 100 person-years of follow-up. The type of immunologic failure according to baseline CD4 count strata in the study population is showed in Table S4. Median time to failure was shorter in patients with baseline CD4>500 cells/µL than with ≤200 cells/µL (8.2 months vs 67.5; P<0.001) (Figure 1C). The results of univariate proportional hazards regression analyses to identify factors associated with risk of failure are shown in table S3. The only statistically significant factors were baseline CD4 counts and VL. All other factors examined, including demographic characteristics and composition of initial regimen by drug class were not significant in the univariate analysis. In the multivariable analysis three factors were included: sex, baseline CD4 counts and VL. The risk of failure was higher for patients with higher baseline CD4 counts (AHR: 1.30; 95% CI: 1.10–1.53; P = 0.002) (Table S3) and lower for patients with baseline VL ≥10,000 copies/mL (AHR: 0.33; 95% CI: 0.12–0.91; P = 0.033).

Among the 60 patients with available data at month 24, median CD4 slopes were uniform across CD4 strata (gain of 6.9 cells/µL/month [3.2–10.6]), with the exception of individuals with baseline >500 cells/µL that lost a median of 7 CD4 cells/µL/month (Table S5). Median TLC slopes were 4.4 cells/µL/month (−1.8; 20.0), while median CD8 slopes were −2.3 cells/µL/month (−10.6; 8.9). A positive correlation was observed between CD4 and TLC slopes (r = 0.669; P<0.001), and CD4 and CD8 slopes (r = 0.452; P = 0.001) (Figure S3).

### Virologic Suppression is Achieved in the Majority of Patients with Available Viral Load Measurements

**Figure 2:**
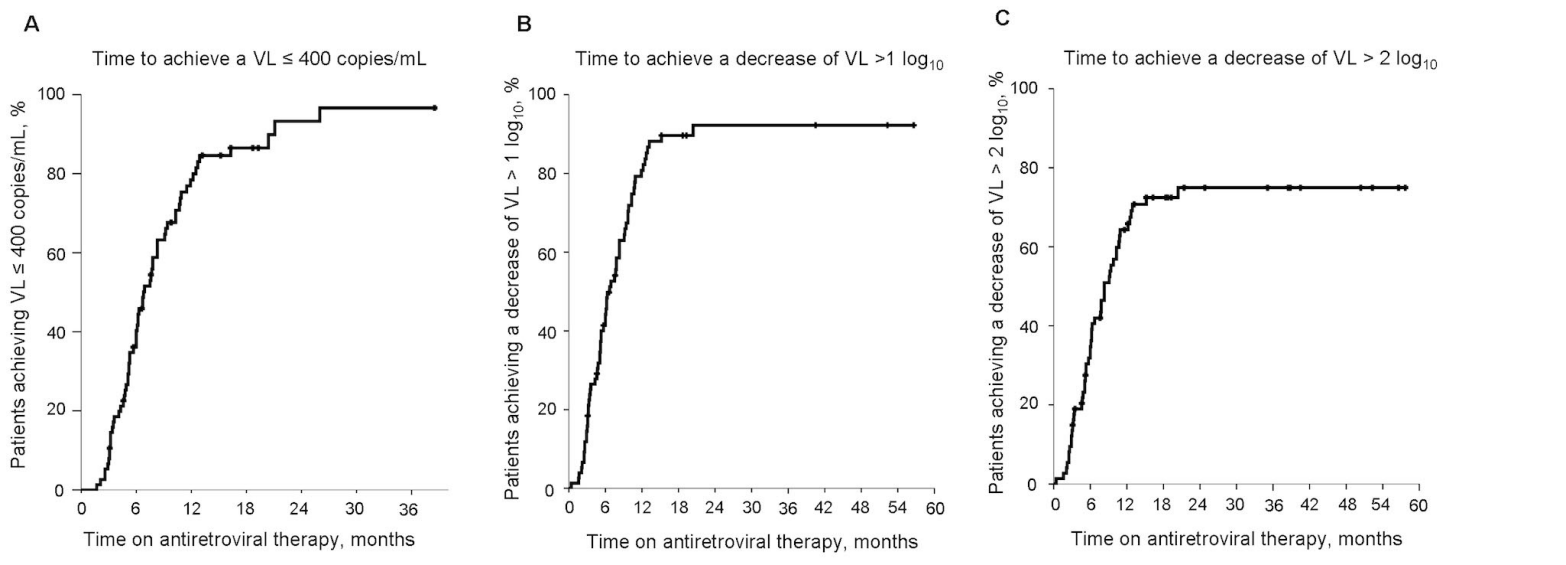
Kaplan-Meier plots of the probability of virologic response during follow-up. Legend: A) time spent on HAART to achieve a HIV-1 RNA (VL) ≤400, copies/mL; B) time spent on HAART to achieve a decrease of HIV-1 RNA (VL) >1 log_10_; C) time spent on HAART to achieve a decrease of HIV-1 RNA (VL) >2 log_10_.

Among patients with available VL, 86% (64/74) experienced a decrease to undetectable VL levels after a median of 6.8 months (5.3–8.4). A VL reduction of >1 log_10_ occurred in 88% and of >2 log_10_ in 69% participants (Figure 2). Resistance testing was performed in 2 patients who failed to suppress viral replication revealing mutations conferring low-to-high resistance to NRTIs (M184V, T215F) and NNRTIs (K101E, G190A) [5]. Among patients who achieved undetectable VL, 8 experienced a rebound after a median time on HAART of 16.5 months (11.2–19.2). One of those had mutations conferring intermediate-to-high resistance to zidovudine and lamivudine (D69N, K70R, M184V).

## Discussion

This is the first study evaluating the predictors of attrition after initiation of HAART as well as HAART effectiveness in a population paying for antiretrovirals and laboratory testing in Mozambique. The retention rate (i.e., patients remaining in care and on ART) was 75%, 48% and 37% after one, two and three years, respectively. Similar high rates of attrition have been previously reported in ART programs in several African countries [6], [7]. In Central Mozambique, one study reported that the proportion of patients retained in care after 12 and 24 months of ART initiation (free ART) was 68% and 59%, respectively [8]. In Maputo, a recent study reported a similarly high lost to follow-up rate (40%) in patients eligible for ART undergoing counselling before treatment [9]. In this study, independent predictors of high attrition rate were attending HCC2 and having low CD4 counts at baseline. Patients who start ART at high CD4 counts (≤500 cells/µL) have decreased risk of progression to AIDS and/or death and increased likelihood of immune recovery [10], [11], [12], [13], [14]. Thus, high morbidity and mortality may account, at least in part, for the high rate of attrition observed in our patients with lower CD4 counts [7]. Having low CD4 counts was also described as predictor of attrition in a large HIV cohort study in India [15]. In addition, the high cost of antiretrovirals and laboratory tests required for HIV management might have increased patient-driven treatment interruptions and limited laboratory testing by some of our patients, especially those attending HCC2 which serves a wider and more diverse population across Maputo compared to HCC1. Similar financial constraints to acquire antiretroviral drugs and laboratory testing have been reported in patients attending the private sector in Kenya and Uganda [2], [3]. Further studies are required to identify the causes for low retention rates in patients with low CD4 counts and to better understand the association between healthcare setting and attrition rate.

A sustained immunologic response was observed, with most patients experiencing CD4 recovery above 200 cells/µL, a clinically meaningful threshold for clinical progression and opportunistic infections (OI) [16], [17]. Nevertheless, the recovery was slow and only 46% of individuals reached CD4≥500 cells/µL, similarly to the outcome reported by other surveys performed in high-income and resource-constrained countries [18], [19]. A biphasic kinetic of peripheral blood CD4 T-cell recovery was observed, characterized by a rapid average increase during the first semester followed by a slower increase thereafter. The magnitude of initial immunologic improvements attributable to HAART is consistent with results of previous longitudinal studies and might be primarily attributed to memory T-cell release from lymphoid tissues [20], [21]. The results are also in line with previous studies performed in Mozambique on patients treated for free under the national ART program [22], [23].

As previously found in the EuroSIDA survey, patients with higher baseline CD4 counts were at higher risk of immunological failure than patients with lower CD4. One reason for this finding could lie in the lower rates of ART adherence that individuals with higher baseline CD4 may have had because at lower risk of OI [24]. This is consistent with the finding that median CD4 at the time of immunological failure (520 cells/µL) was above the threshold of increased OI risk. Interestingly, low baseline VL was also an independent predictor of immunological failure. This finding might be explained by the inverse correlation between VL and CD4 at baseline. Thus, patients with low VL levels and/or high CD4 counts might have inadequate adherence to ART. Future studies are needed to measure adherence levels in this population and its relationship to therapy failure.

The main shortcoming of this study was the limited size of the study population and the difficulty in retaining patients in care might have led to a survivor effect. Notwithstanding these limitations and despite the fact that improved strategies are clearly required to increase in-care retention levels, this study suggests that HAART can be effective in HIV-1 infected patients from Mozambique that pay for their medication and laboratory testing. Further studies are required to identify the causes for low retention rates in patients with low CD4 counts and to better understand the association between healthcare setting and attrition rate.

## Supporting Information

Figure S1 **Kaplan-Meier estimate of time from antiretroviral therapy initiation to attrition during follow-up according to healthcare centers.**
(TIF)

Figure S2 **Evolution of immunologic and biochemical parameters during follow-up in HIV-1-infected patients.** Legend: Dots represent median values over the study period; bars represent interquartile range.
(TIFF)

Figure S3 **Median changes in total lymphocytes, CD4 and CD8 counts per month during follow-up.** Legend: Slopes were computed by Deming regression analysis; correlation between slopes was assessed by Spearman Rank test. TLC, total lymphocyte count.
(TIFF)

Table S1 **Comparison of baseline characteristics and antiretroviral therapy use among HIV-1 infected patients retained in the cohort and those not retained.**
(DOC)

Table S2 **Evolution of CD4 T-cell count at different time points during follow-up in the study population.**
(DOC)

Table S3 **Analyses of risk of immunologic failure in the study population.**
(DOC)

Table S4 **Type of immunological failure according to baseline CD4 count strata in the study population.**
(DOC)

Table S5 **Slopes of CD4 count during the first two years of antiretroviral therapy in the study population.**
(DOC)
