# Supplementary figures and images for: Predictors of Attrition and Immunological Failure in HIV-1 Patients on Highly Active Antiretroviral Therapy from Different Healthcare Settings in Mozambique
Source: PLoS One. 2013 Dec 20;8(12):e82718. doi: 10.1371/journal.pone.0082718 (PMC3869714; doi:10.1371/journal.pone.0082718)

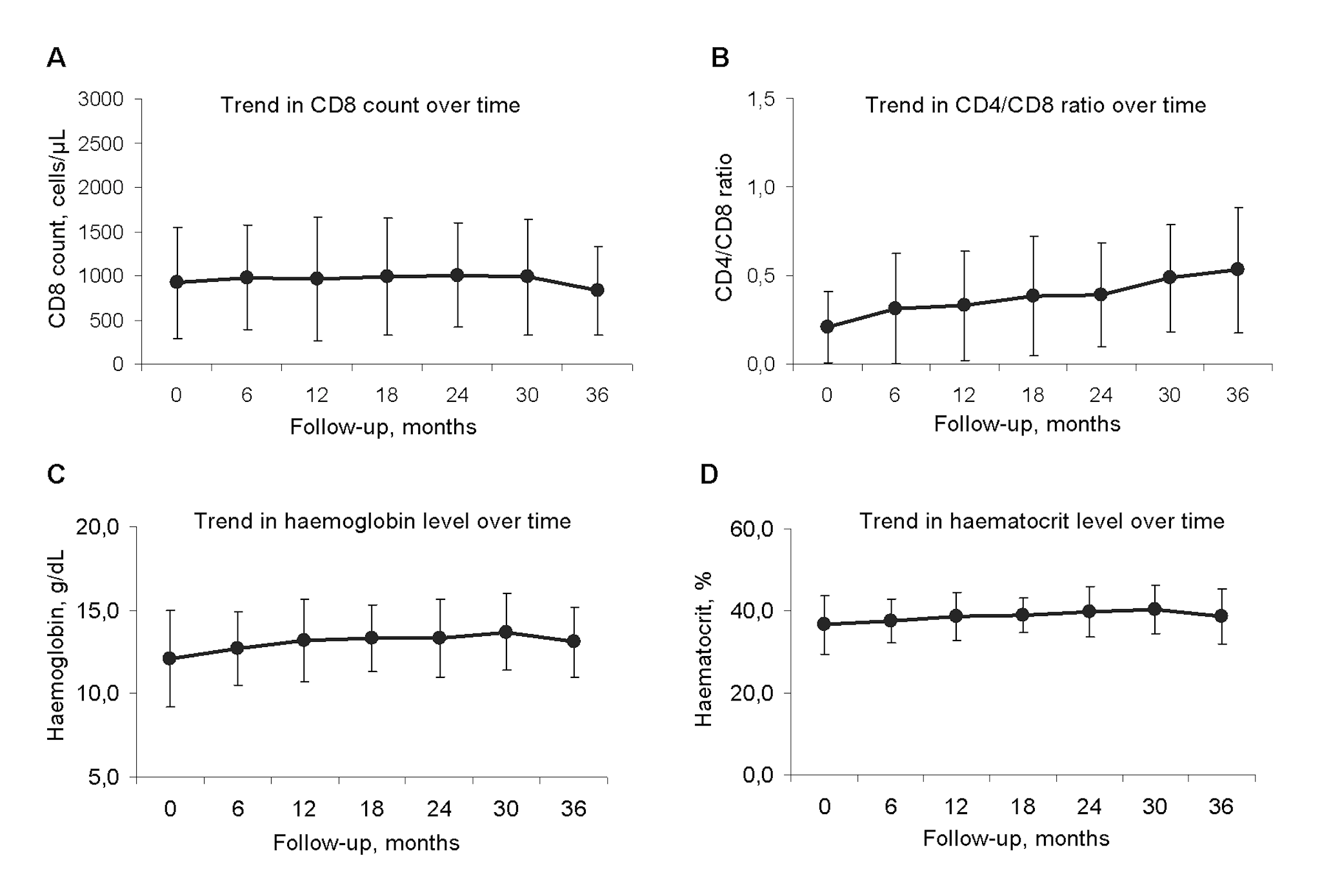

Supplement: Figure S2 — Evolution of immunologic and biochemical parameters during follow-up in HIV-1-infected patients. Legend: Dots represent median values over the study period; bars represent interquartile range. (TIFF) [file pone.0082718.s002.tiff]

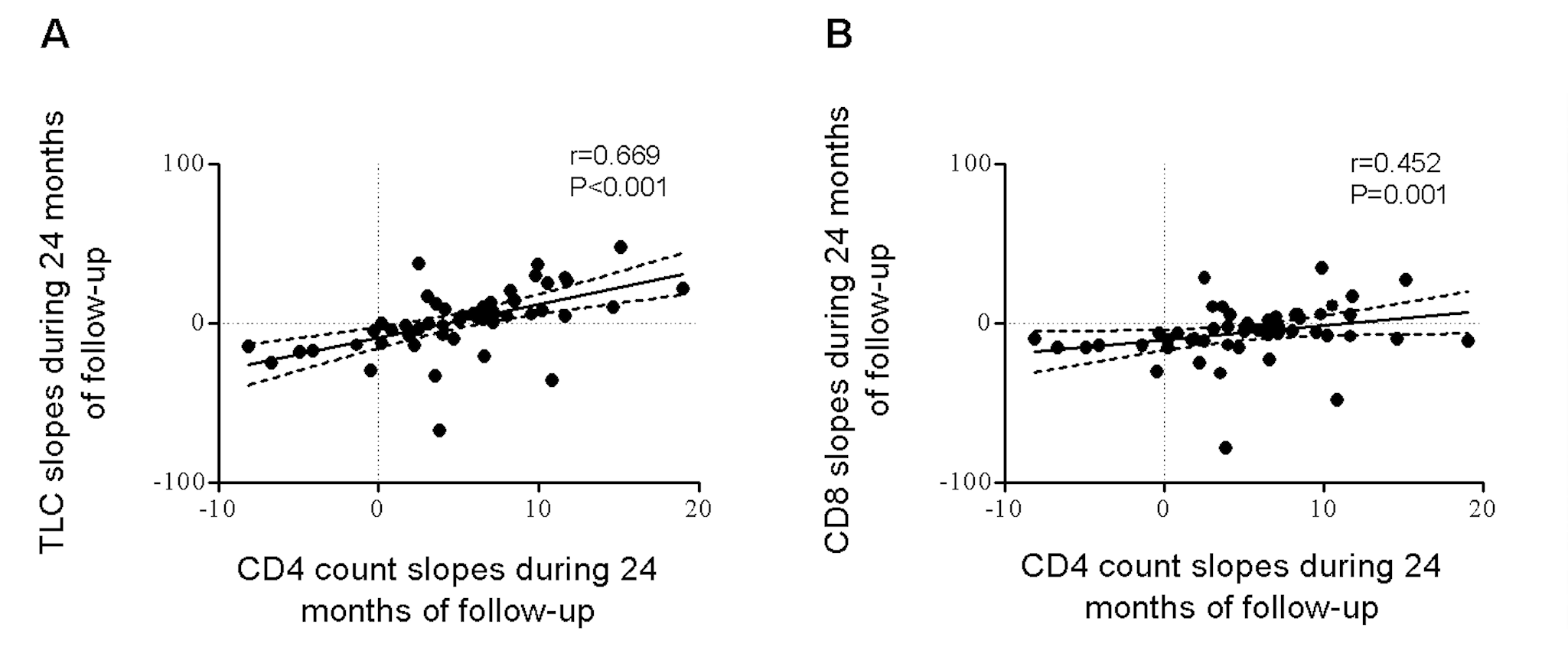

Supplement: Figure S3 — Median changes in total lymphocytes, CD4 and CD8 counts per month during follow-up. Legend: Slopes were computed by Deming regression analysis; correlation between slopes was assessed by Spearman Rank test. TLC, total lymphocyte count. (TIFF) [file pone.0082718.s003.tiff]
