# Supplementary material for: Predictors of Attrition and Immunological Failure in HIV-1 Patients on Highly Active Antiretroviral Therapy from Different Healthcare Settings in Mozambique
Source: PLoS One. 2013 Dec 20;8(12):e82718. doi: 10.1371/journal.pone.0082718 (PMC3869714; doi:10.1371/journal.pone.0082718)
Supplement: Table S1 — Comparison of baseline characteristics and antiretroviral therapy use among HIV-1 infected patients retained in the cohort and those not retained. (DOC) [file pone.0082718.s004.doc]

**Supporting Information Table 1. Comparison of baseline characteristics and antiretroviral therapy use among HIV-1 infected patients retained in the cohort and those not retained.**

|  | ***All patients enrolled*** | ***Patients retained in care*** | ***Patients not retained in care*** |  |
| --- | --- | --- | --- | --- |
| ***Baseline characteristics*** | ***(N= 142)*** | ***(N=52 )*** | ***(N=90 )*** | ***P*** |
| Age in years ^a^ | 39.5 (31.0-46.0) | 39.0 (32.0-47.8) | 40.0 (30.8-46.0) | .50 |
| Sex  ^b^ |  |  |  |  |
| Men | 82 (57.7) | 32 (39.0) | 50 (61.0) | .60 |
| Women | 60 (42.3) | 20 (33.3) | 40 (66.7) |  |
| Ethnicity ^b^ |  |  |  |  |
| Black | 108 (76.1) | 39 (36.1) | 69 (63.9) | .51 |
| Others * | 27 (19.0) | 12 (44.4) | 15 (55.6) |  |
| Unknown | 7 (4.9) | 1 (14.3) | 6 (85.7) |  |
| Immune category ^b^ |  |  |  |  |
| >500 cells/µL | 7 (4.9) | 4 (57.1) | 3 (42.9) | ***.049*** |
| 201-500 cells/µL | 57 (40.1) | 26 (45.6) | 31 (54.4) |  |
| ≤200 cells/µL | 78 (54.9) | 22 (28.2) | 56 (71.8) |  |
| Lymphocyte count ^a^ |  |  |  |  |
| TLC, cells/μL | 1,243 (936-1,681) | 1,350 (1,027-1,783) | 1,196 (861-1,635) | .08 |
| CD4 count, cells/μL | 185 (126-290) | 248 (153-329) | 170 (111-248) | ***.005*** |
| CD4 % | 16.0 (10.0-23.0) | 17.0 (11.0-22.8) | 14.0 (7.8-23.0) | .16 |
| CD8 count, cells/μL | 920 (672-1,313) | 1,006 (817-1,327) | 860 (608-1,307) | .07 |
| CD8 % | 77.0 (69.0-85.0) | 75.5 (71.0-84.0) | 77.0 (67.0-85.3) | .88 |
| CD4/CD8 ratio | 0.21 (0.12-0.32) | 0.23 (0.13-0.32) | 0.19 (0.09-0.33) | .22 |
| Laboratory data ^a^ (N=121‡) |  |  |  |  |
| White blood cell, % | 4.4 (3.6-6.0) | 4.5 (3.8-6.1) | 4.4 (3.5-6.0) | .64 |
| Erythrocytes, % | 4.0 (3.6-4.6) | 4.1 (3.8-4.7) | 4.0 (3.5-4.6) | .45 |
| Platelet count, cells/μL | 215 (176-267) | 232 (159-286) | 210 (176-255) | .50 |
| Haemoglobin, g/dL | 12.1 (10.7-13.6) | 12.3 (11.0-13.3) | 11.9 (10.5-13.7) | .62 |
| Haematocrit, % | 36.6 (33.3-40.6) | 37.2 (34.7-40.5) | 36.6 (32.7-40.6) | .39 |
| Plasma HIV-1 RNA ^a^ (N=76†) |  |  |  |  |
| HIV-1 RNA, copies/mL | 213,000 (60,275-755,000) | 318,500 (27,200-1,010,000) | 199,000 (82,150-754,000) | .85 |
| log_10_ HIV-1 RNA, copies/mL | 5.3 (4.8-5.9) | 5.5 (4.4-6.0) | 5.3 (4.9-5.9) | .85 |
| **Antiretroviral use** |  |  |  |  |
| Months of ART ^a^ | 22.2 (12.1-46.7) | 51.2 (36.9-58.9) | 16.4 (7.8-21.7) | ***<.001*** |
| Switch of regimen |  |  |  |  |
| One switch ^b^ | 5 (3.5) | 3 (60.0) | 2 (40.0) |  |
| Months of ART before switch ^a^ | 12.2 (8.4-14.4) | 8.2; 12.2; 16.4 | 8.7; 12.3 | N/A |
| Two switches ^b^ | 2 (1.4) | 1 (50.0) | 1 (50.0) |  |
| Months of ART before switch | 18.2 | 17.7 | 18.7 |  |
| Single drug substitution  ^b^ | 10 (7.0) | 6 (60.0) | 4 (40.0) |  |
| First-line regimen ^b^ |  |  |  |  |
| 2 NRTI + 1 NNRTI | 125 (88.0) | 44 (35.2) | 81 (64.8) | .77 ** |
| d4T+3TC+NVP | 43 (34.4) | 16 (37.2) | 27 (62.8) |  |
| AZT+3TC+NVP | 35 (28.0) | 12 (34.3) | 23 (65.7) |  |
| 2 NRTI + 1 PI | 14 (9.9) | 6 (42.9) | 8 (57.1) |  |
| Other ^c^ | 3 (2.1) | 2 (66.7) | 1 (33.3) |  |
| Second-line regimen ^b^ (N=7) |  |  |  |  |
| 2 NRTI + 1 PI | 6 (85.7) | 4 (66.7) | 2 (33.3) |  |
| 2 NRTI + 1 NNRTI | 1 (14.3) | -- | 1 (100.0) |  |

Legend: ^a^ median (interquartile range, IQR); ^b^ number (%); ^c^ includes two patients treated with 2NRTI + 1 boosted-PI and one patient with 1NRTI + 1NNRTI + 1PI; * includes 17 Whites, 9 Black/White and 1 Asian; ‡ includes 69 men and 52 women; † includes 47 men and 29 women; TLC, Total lymphocyte count; ART, antiretroviral therapy; ART, antiretroviral therapy; NRTI, nucleoside reverse transcriptase inhibitor; NNRTI, non-nucleoside reverse transcriptase inhibitor; PI, protease inhibitor; AZT, zidovudine; d4T, stavudine; 3TC, lamivudine; NVP, nevirapine; N/A, NA, not applicable. Percentages were compared between patients in care and those lost-to-follow up using Fisher exact test or χ2 as appropriate and medians were compared by Mann-Whitney U test (*P*<0.05); ** comparison between 2 NRTI + 1 NNRTI regimen vs 2 NRTI + 1 PI regimen.
