# Supplementary material for: Predictors of Attrition and Immunological Failure in HIV-1 Patients on Highly Active Antiretroviral Therapy from Different Healthcare Settings in Mozambique
Source: PLoS One. 2013 Dec 20;8(12):e82718. doi: 10.1371/journal.pone.0082718 (PMC3869714; doi:10.1371/journal.pone.0082718)
Supplement: Table S2 — Evolution of CD4 T-cell count at different time points during follow-up in the study population. (DOC) [file pone.0082718.s005.doc]

**Supporting Information Table 2. Evolution of CD4 T-cell count** **at different time points during follow-up in the study population.**

|  | **Month 6** | | | | | **Month 12** | | | | |
| --- | --- | --- | --- | --- | --- | --- | --- | --- | --- | --- |
| **Baseline CD4 count strata** (cells/μL) | **N (%)** | **Median CD4 count** (cells/μL, IQR) | **Median increase relative to baseline** (cells/μL, IQR) | **≤350** N (%) | **>350**  N (%) | **N (%)** | **Median CD4 count** (cells/μL,  IRQ) | **Median increase relative to baseline** (cells/μL, IQR) | **≤350** N (%) | **>350** N (%) |
| All | 102 (71.8) | 313 (201 ; 419) | 113 (38 ; 187) | 65 (63.7) | 37 (36.3) | 90 (63.4) | 334 (236 ; 460) | 127 (25 ; 248) | 47 (52.2) | 43 (47.8) |
| ≤200 | 58 (74.4) | 233 (162 ; 323) | 106 (51 ; 181) | 51 (87.9) | 7 (12.1) | 52 (66.7) | 279 (169 ; 373) | 135 (23 ; 242) | 37 (71.2) | 15 (28.8) |
| 201-350 | 28 (65.1) | 378 (289 ; 463) | 116 (43 ; 227) | 11 (39.3) | 17 (60.7) | 27 (62.8) | 420 (324 ; 516) | 151 (63 ; 292) | 8 (29.6) | 19 (70.4) |
| 351-500 | 10 (71.4) | 510 (351 ; 577) | 121 (-55 ; 174) | 2 (20.0) | 8 (80.0) | 7 (50.0) | 510 (381 ; 560) | 106 (-12 ; 163) | 1 (14.3) | 6 (85.7) |
| >500 | 6 (85.7) | 690 (388 ; 972) | -89 (-133 ; 294) | 1 (16.7) | 5 (83.3) | 4 (57.1) | 538 (296 ; 681) | -148 (-289 ; -12) | 1 (25.0) | 3 (75.0) |

**Supporting Information Table 2. Continue**

|  | **Month 24** | | | | | **Month 36** | | | | |
| --- | --- | --- | --- | --- | --- | --- | --- | --- | --- | --- |
| **Baseline CD4 count strata** (cells/μL) | **N (%)** | **Median CD4 count** (cells/μL, IQR) | **Median increase relative to baseline** (cells/μL, IQR) | **≤350** N (%) | **>350** N (%) | **N (%)** | **Median CD4 count** (cells/μL, IRQ) | **Median increase relative to baseline** (cells/μL, IQR) | **≤350** N (%) | **>350** N (%) |
| All | 62 (43.7) | 372 (271 ; 491) | 164 (73 ; 252) | 27 (43.5) | 35 (56.5) | 52 (36.6) | 451 (344 ; 571) | 209 (84 ; 360) | 14 (26.9) | 38 (73.1) |
| ≤200 | 35 (44.9) | 300 (216 ; 381) | 163 (83 ; 274) | 22 (62.9) | 13 (37.1) | 22 (28.2) | 364 (262 ; 508) | 245 (94 ; 375) | 10 (45.5) | 12 (54.5) |
| 201-350 | 18 (41.9) | 467 (365 ; 590) | 215 (109 ; 351) | 4 (22.2) | 14 (77.8) | 20 (46.5) | 519 (413 ; 685) | 267 (163 ; 415) | 3 (15.0) | 17 (85.0) |
| 351-500 | 5 (35.7) | 482 (443 ; 587) | 130 (-1 ; 174) | -- | 5 (100.0) | 6 (42.9) | 464 (332 ; 705) | 90 (-66 ; 240) | 1 (16.7) | 5 (83.3) |
| >500 | 4 (57.1) | 582 (302 ; 732) | -172 (-304 ; 129) | 1 (25.0) | 3 (75.0) | 4 (57.1) | 559 (450 ; 950) | -29 (-85 ; 61) | -- | 4 (100) |
