# Supplementary material for: Predictors of Attrition and Immunological Failure in HIV-1 Patients on Highly Active Antiretroviral Therapy from Different Healthcare Settings in Mozambique
Source: PLoS One. 2013 Dec 20;8(12):e82718. doi: 10.1371/journal.pone.0082718 (PMC3869714; doi:10.1371/journal.pone.0082718)
Supplement: Table S3 — Analyses of risk of immunologic failure in the study population. (DOC) [file pone.0082718.s006.doc]

**Supporting Information Table 3. Analyses of risk of immunologic failure in the study population.**

|  | **Immunologic failure** | | | |
| --- | --- | --- | --- | --- |
| **Analysis, factor** | **N.** | **N. of cases (%)** | **Hazard ratio (95% CI)** | **P** |
| **Univariate** |  |  |  |  |
| Age at first regimen start ^a^ | 142 | 46 (32.4) | 1.03 (0.89-1.18) | .73 |
| Sex |  |  |  |  |
| Men | 82 | 26 (31.7) | 1 |  |
| Women | 60 | 20 (33.3) | 1.36 (0.76-2.44) | .31 |
| Ethnicity |  |  |  |  |
| Other | 27 | 7 (25.9) | 1 |  |
| Black | 108 | 36 (33.3) | 1.39 (0.62-3.13) | .43 |
| Baseline CD4 count ^b^ | 142 | 46 (32.4) | 1.30 (1.11-1.50) | *.001* |
| Baseline log_10_ HIV-1 RNA |  |  |  |  |
| <10,000 | 8 | 5 (62.5) | 1 |  |
| ≥10,000 | 68 | 19 (27.9) | 0.26 (0.10-0.72) | *.009* |
| Unknown | 66 | 22 (33.3) | 0.29 (0.11-0.79) | .*015* |
| First-line regimen ^c^ |  |  |  |  |
| PI-based | 16 | 6 (37.5) | 1 |  |
| NNRTI-based | 125 | 39 (31.2) | 0.84 (0.36-1.99) | .70 |
| **Multivariable** |  |  |  |  |
| Sex |  |  |  |  |
| Men | 82 | 26 (31.7) | 1 |  |
| Women | 60 | 20 (33.3) | 1.42 (0.78-2.58) | .25 |
| Baseline CD4 count ^b^ | 142 | 46 (32.4) | 1.30 (1.10-1.53) | *.002* |
| Baseline log_10_ HIV-1 RNA |  |  |  |  |
| <10,000 | 8 | 5 (62.5) | 1 |  |
| ≥10,000 | 68 | 19 (27.9) | 0.33 (0.12-0.91) | *.033* |
| Unknown | 66 | 22 (33.3) | 0.35 (0.13-0.97) | .*044* |

Legend: Adjusted hazard ratios (AHR) were derived from a standard Cox proportional hazard model. CI, confidence intervals; NNRTI, nonnucleoside reverse-transcriptase inhibitor; PI, protease inhibitor.

ª Per 5-y increase, ^b^ per 100 cells/μL, ^c^ excludes one patient who was treated with 1NRTI + 1NNRTI + 1PI as initial HAART regimen.
