# Supplementary material for: Predictors of Attrition and Immunological Failure in HIV-1 Patients on Highly Active Antiretroviral Therapy from Different Healthcare Settings in Mozambique
Source: PLoS One. 2013 Dec 20;8(12):e82718. doi: 10.1371/journal.pone.0082718 (PMC3869714; doi:10.1371/journal.pone.0082718)
Supplement: Table S4 — Type of immunological failure according to baseline CD4 count strata in the study population. (DOC) [file pone.0082718.s007.doc]

**Supporting Information Table 4. Type of immunologic failure according to baseline CD4 count strata in the study population.**

| **Immunological failure** | | | | | | | | | |
| --- | --- | --- | --- | --- | --- | --- | --- | --- | --- |
|  | **All study period** | **Month 12** | | | | **Month 24** | | | |
| **Baseline CD4 count strata** (cells/μL) | **N (%)** | **N (%)** | **Fall of CD4 count to baseline** | **50% fall from on-treatment peak value** | **Persistent CD4 count <100 cells/μL** | **N (%)** | **Fall of CD4 count to baseline** | **50% fall from on-treatment peak value** | **Persistent CD4 count <100 cells/μL** |
| **All** | **46** | **29** | **29** | **9** | **5** | **10** | **8** | **4** | **1** |
| ≤200 | 21 (45.7) | 14 (48.3) | 14 (48.3) | 4 (44.4) | 5 (100.0) | 5 (50.0) | 3 (37.5) | 3 (0.75) | 1 (100.0) |
| 201-350 | 11 (23.9) | 6 (20.7) | 6 (20.7) | 1 (11.1) | -- | 3 (30.0) | 3 (37.5) | -- | -- |
| 351-500 | 8 (17.4) | 4 (13.8) | 4 (13.8) | -- | -- | 1 (10.0) | 1 (12.5) | 1 (0.25) | -- |
| >500 | 6 (13.0) | 5 (17.2) | 5 (17.2) | 4 (44.4) | -- | 1 (10.0) | 1 (12.5) | -- | -- |

**Supporting Information Table 4. Continue**

| **Immunological failure** | | | | | | | | | |
| --- | --- | --- | --- | --- | --- | --- | --- | --- | --- |
|  | **All study period** | **Month 36** | | | | **Month >36** | | | |
| **Baseline CD4 count strata**  (cells/μL) | **N (%)** | **N (%)** | **Fall of CD4 count to baseline** | **50% fall from on-treatment peak value** | **Persistent CD4 count <100 cells/μL** | **N (%)** | **Fall of CD4 count to baseline** | **50% fall from on-treatment peak value** | **Persistent CD4 count <100 cells/μL** |
| **All** | **46** | **2** | **1** | **1** | **--** | **5** | **4** | **1** | **--** |
| ≤200 | 21 (45.7) | 1 (50.0) | -- | 1 (100.0) | -- | 1 (20.0) | 1 (25.0) | -- | -- |
| 201-350 | 11 (23.9) | -- | -- | -- | -- | 2 (40.0) | 1 (25.0) | 1 (100.0) | -- |
| 351-500 | 8 (17.4) | 1 (50.0) | 1 (100.0) | -- | -- | 2 (40.0) | 2 (50.0) | -- | -- |
| >500 | 6 (13.0) | -- | -- | -- | -- | -- | -- | -- | -- |

Legend: Immunological failure categories are not exclusive, thus the same patient might be included in more than one category.
