# Supplementary material for: Predictors of Attrition and Immunological Failure in HIV-1 Patients on Highly Active Antiretroviral Therapy from Different Healthcare Settings in Mozambique
Source: PLoS One. 2013 Dec 20;8(12):e82718. doi: 10.1371/journal.pone.0082718 (PMC3869714; doi:10.1371/journal.pone.0082718)
Supplement: Table S5 — Slopes of CD4 count during the first two years of antiretroviral therapy in the study population. (DOC) [file pone.0082718.s008.doc]

**Supporting Information Table 5. Slopes of CD4 count during the first two years of antiretroviral therapy in the study population.**

| **CD4 count** (cells/μL) | **N (%)** | **Median CD4 count change** (cells/μL/month) | **P** |
| --- | --- | --- | --- |
| All | 60 (100.0) | 6.9 (3.2 ; 10.6) | *0.028* |
| ≤200^a^ | 33 (55.0) | 7.0 (3.5 ; 11.6) |  |
| 201-350^b^ | 18 (30.0) | 8.9 (4.5 ; 14.6) |  |
| 351-500 | 5 (8.3) | 5.4 (-0.04 ; 7.2) |  |
| >500^a,b^ | 4 (6.7) | -7.1 (-12.7 ; 5.4) |  |

Legend: Box plots represent median changes of CD4 count (cells/μL) calculated from 3 consecutive measurements by Deming linear regression according to CD4 count strata (≤200; 201-350; 351-500; ≥500). Medians were compared among CD4 strata using Kruskal-Wallis test and Mann-Whitney U test (P<0.05).

^a^ Mann-Whitney U test, P=0.018, ^b^ Mann-Whitney U test, P=0.010.
